# Molecular Characteristics, mRNA Expression, and Alternative Splicing of a Ryanodine Receptor Gene in the Oriental Fruit Fly, *Bactrocera dorsalis* (Hendel)

**DOI:** 10.1371/journal.pone.0095199

**Published:** 2014-04-16

**Authors:** Guo-Rui Yuan, Wen-Zhi Shi, Wen-Jia Yang, Xuan-Zhao Jiang, Wei Dou, Jin-Jun Wang

**Affiliations:** Key Laboratory of Entomology and Pest Control Engineering, College of Plant Protection, Southwest University, Chongqing, China; Institute of Vegetables and Flowers, Chinese Academy of Agricultural Science, China

## Abstract

Ryanodine receptors (RyRs) are a distinct class of ligand-gated channels controlling the release of calcium from intracellular stores. The emergence of diamide insecticides, which selectively target insect RyRs, has promoted the study of insect RyRs. In the present study, the full-length RyR cDNA (*BdRyR*) was cloned and characterized from the oriental fruit fly, *Bactrocera dorsalis* (Hendel), a serious pest of fruits and vegetables throughout East Asia and the Pacific Rim. The cDNA of *BdRyR* contains a 15,420-bp open reading frame encoding 5,140 amino acids with a predicted molecular weight of 582.4 kDa and an isoelectric point of 5.38. *BdRyR* shows a high level of amino acid sequence identity (78 to 97%) to other insect RyR isoforms. All common structural features of the RyRs are present in the BdRyR, including a well-conserved C-terminal domain containing consensus calcium-binding EF-hands and six transmembrane domains, and a large N-terminal domain. Quantitative real-time PCR analyses revealed that *BdRyR* was expressed at the lowest and highest levels in egg and adult, respectively, and that the *BdRyR* expression levels in the third instar larva, pupa and adult were 166.99-, 157.56- and 808.56-fold higher, respectively, than that in the egg. Among different adult body parts, the highest expression level was observed in the thorax compared with the head and abdomen. In addition, four alternative splice sites were identified in the *BdRyR* gene, with the first, ASI, being located in the central part of the predicted second spore lysis A/RyR domain. Diagnostic PCR analyses revealed that alternative splice variants were generated not only in a tissue-specific manner but also in a developmentally regulated manner. These results lay the foundation for further understanding the structural and functional properties of *BdRyR*, and the molecular mechanisms for target site resistance in *B*. *dorsalis*.

## Introduction

Ryanodine, a toxic natural alkaloid, has long been used as an insecticide [1]; however, its mammalian toxicity has limited its use. Recently, two novel classes of synthetic chemicals have emerged, resulting in commercial insecticides targeting the ryanodine receptor (RyR). The first class is the phthalic acid diamides, which includes flubendiamide [2]. The second class is the anthranilic diamides, which includes chlorantraniliprole [3]. These novel synthetic chemicals are potent activators of insect RyRs [4] and are particularly useful against those pest species that have developed severe resistance to other chemical classes of insecticides [5].

Calcium is a universal intracellular messenger, and its release from intracellular stores is essential for the initiation and propagation of many Ca^2+^ signaling events such as contraction, and hormone and neurotransmitter release [6], [7]. In mammalian cells, the Ca^2+^ release is mediated by at least two classes of channel proteins, the inositol 1,4,5-trisphosphate (IP3) receptor and the RyR [6]. As the largest ion channel currently known, the RyR is an approximately 2-MDa homotetramer. Each monomer consists of more than 5,000 amino acids, including the carboxyl-terminal channel region, which has four to 12 putative transmembrane segments, and the remaining portion, the “foot”, which spans the junction gap between the transverse tubules and sarcoplasmic reticulum membranes [8], [9]. It has been proposed that the specific interaction between the cytoplasmic and transmembrane domains is an important mechanism in the intrinsic modulation of the RyR [10].

To date, three isoforms of RyR proteins are known to be encoded by different genes in mammals. RyRs are expressed in many tissues, but their expression patterns are various. Previous research showed the localization of RyRs: RyR1 primarily to skeletal muscle, RyR2 to heart and brain, and RyR3 to brain and some peripheral tissues. Subsequent studies found that all the RyRs were widely expressed in brain and peripheral tissues [11]–[15]. They are similar in their primary structure (showing 66% identical sequence), except for three regions with high degrees of variability [16]. In birds, amphibians and fish, two RyRs, RyRA and RyRB are homologous to mammalian RyR1 and RyR3, respectively [17]–[19]. In contrast to mammals, only one gene encoding a RyR is present in *Drosophila melanogaster*, and it shares a 44 to 46% amino acid identity with the RyR mammalian isoforms [20]. Interestingly, mammalian and insect RyRs are more different in sequence than their corresponding inositol trisphosphate receptors (InsP3Rs), which may make RyRs better targets for insecticidal molecules with low mammalian toxicity. Recently, cDNAs encoding ryanodine receptors were cloned and characterized from a series of pest insects, including *Bombyx mori* (*sRyR*), *Plutella xylostella* (*PxRyR*), *Cnaphalocrocis medinalis* (*CmRyR*), *Nilaparvata lugens* (*NlRyR*), *Ostrinia furnacalis* (*OfRyR*), *Helicoverpa armigera* (*HaRyR*), and *Pieris rapae* (*PrRyR*) [21]–[27]. With extensive and repetitive applications of diamide insecticides, a low-level resistance, about 9-fold, was first reported in field populations of *P. xylostella* in China [28]. Subsequently, high levels of resistance to these chemicals evolved in some field populations of *P. xylostella* from the Philippines, Thailand and China [29]–[31]. Studies on mechanisms of resistance to diamide insecticides in the *P. xylostella* have been performed by several labs, and, to some extent, metabolic detoxification was involved in chlorantraniliprole resistance in field populations of *P. xylostella* [31]. Additionally, a single amino acid substitution (G4946E) in the C-terminal membrane-spanning domain of the RyR was reported to be associated with high levels of diamide resistance in field *P. xylostella* populations collected from the Philippines and Thailand [30]. This was confirmed using ligand binding assays [32].

The oriental fruit fly, *Bactrocera dorsalis* (Hendel) (Diptera: Tephritidae), is a serious pest of fruits and vegetables throughout East Asia and the Pacific Rim [33]. It damages various fruits by ovipositing inside them, where the larvae feed until pupation, which causes fruit drop and a loss in fruit value [34]. Among many control measures, spraying chemical insecticides has been the primary strategy for controlling *B. dorsalis*. With extensive exposure to insecticides in the field, *B. dorsalis* has evolved resistance to a range of insecticides, including organophosphate, pyrethroid, carbamate, and avermectin [35], [36]. Novel chemicals that act on new targets with no cross-resistance to the current insecticides are thus needed to cope with *B. dorsalis* resistance. To date, there have been no published reports of resistance in *B. dorsalis* to the two classes of diamides. However, with the application of these chemicals for *B. dorsalis* control, the problem of resistance may occur in the future. To carry out further studies focused on understanding the mechanism of target site resistance to diamides in *B. dorsalis*, we isolated and characterized its full-length RyR cDNA (*BdRyR*). Additionally, we investigated the mRNA expression pattern and developmentally regulated alternative splicing of *BdRyR*.

## Materials and Methods

### Ethics statement

No specific permits were required for the insects collected in this study. The sampling locations were not privately owned or protected in any way, and the collection did not involve endangered or protected species.

### Insects

The laboratory colony of *B. dorsalis* was originally collected in 2009 from Fuzhou in Fujian Province, China. They were reared in the laboratory at 27±1°C and 70±5% relative humidity with a photoperiod of 14∶10 (L: D). The eggs, larvae, pupae, and adults were collected, immediately frozen in liquid nitrogen, and stored at −80°C for further use.

### Polymerase chain reaction (PCR) amplification and rapid amplification of cDNA ends (RACE)

**Figure 1:**
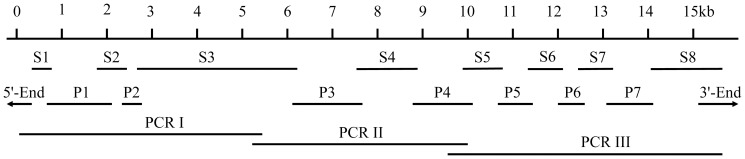
PCR amplification and cloning of the ryanodine receptor (*RyR*) cDNA in the oriental fruit fly, *Bactrocera dorsalis* (Hendel). Eight PCR fragments (S1 to S8) were generated from a transcriptome sequencing database of *B. dorsalis*. Based on these sequences, seven gaps (P1 to P7) were amplified. The 5'- and 3'-end fragments were obtained through 5'- and 3'-RACE, respectively. PCRI to III fragments were amplified with specific primers designed according to the assembled full-length cDNA sequences of *BdRyR*.

Total RNAs from eggs, larvae, pupae, adults, and each body part of adult *B. dorsalis* were isolated using a TRIzol kit (Invitrogen, Carlsbad, CA, USA), and first strand cDNA was synthesized from 2 µg of each total RNA preparation using an oligo (dT)_15_ primer and SuperScript III reverse transcriptase (Invitrogen) according to the manufacturer's instructions. Eleven short cDNA fragments encoding *BdRyR* were identified from a transcriptome sequencing data of *B. dorsalis* [37], and eight fragments (S1 to S8) were assembled by sequence alignment according to the *RyR* sequence of *D. melanogaster* (Table 1, Figure 1). Based on the obtained sequences, seven pairs of primers (Table 2) were designed using Primer Premier 5.0 (Premier Biosoft International, Palo Alto, CA, USA) to amplify the seven gaps between the eight assembled fragments of *BdRyR*. The 5'- and 3'-RACE reactions were performed using the SMARTer™ RACE cDNA Amplification Kit (Clontech, Palo Alto, CA, USA) following the instructions of the manufacturer. The cDNA fragments from PCR amplifications were cloned into a pGEM-T Easy vector (Promega, Madison, WI, USA) and sequenced using an ABI Model 3100 automated sequencer (Life Technologies, Shanghai, China). To validate the reliability of the assembled full-length cDNA sequences of BdRyR, three pairs of primers (Table 2) were designed to amplify three overlapping cDNA fragments (PCRI to PCRIII, Figure 1). LA Taq polymerase (TaKaRa, Dalian, China) was used for long-distance PCR amplifications. The PCR products (≈5 Kb) were cloned into plasmid pCR-XL-TOPO (Invitrogen) and then sequenced.

**Table 1:** *Bactrocera dorsalis*' ryanodine receptor (*BdRyR*) cDNA fragments extracted from a transcriptome sequencing database.

cDNA fragment	Length (bp)	Position in the full length cDNA of *BdRyR* (bp)
**S1**	423	451–873
**S2**	516	1903–2417
**S3**	3627	2648–6274
**S4**	1325	7519–8843
**S5**	848	9924–10 771
**S6**	765	11 320–12 084
**S7**	704	12 514–13 217
**S8**	1583	14 063–15 645

**Table 2:** Primers used in molecular cloning of *Bactrocera dorsalis*' ryanodine receptor cDNA and diagnostic PCR.

Primer name	Primer sequence (5'-3')	Description (cDNA position)
**P1-F**	GCAAGAACATAGCCAAGGTGAG	RT-PCR product P1 (600–2181)
**P1-R**	TCCATCTACTTGACCAACAAAAATA	
**P2-F**	GGGGAGGAAACGGTGTAGGTGAT	RT-PCR product P2 (2318–2810)
**P2-R**	GAAGAAGGAAGTGCGACACCAGAAG	
**P3-F**	TACGACATACAACTGCGGCATAGGG	RT-PCR product P3 (6238–7559)
**P3-R**	GGACGGCGGATAAGAAGACGAATGA	
**P4-F**	GAAAGTGGATGGTCCTATGGCGAGA	RT-PCR product P4 (8815–10 076)
**P4-R**	TGTGTGAGCGTTCCATCAGTTCCTA	
**P5-F**	TTGTTCGGGACATTTATTCG	RT-PCR product P5 (10 739–11 365)
**P5-R**	CTTGCGTCTTGTCCTTGCCTTTATC	
**P6-F**	TGGGAATAGCAATACTCCGTGGTGG	RT-PCR product P6 (12 011–12 594)
**P6-R**	GAAACCCCCAACAGCGTCCCATAAA	
**P7-F**	GTTTAGCCCGATTTTTAGAGACAGC	RT-PCR product P7 (13 094–14 169)
**P7-R**	GAGGAAACTAACGGCACGAT	
**RyR-5'-R1**	TTTCTCGCCTTCAGACCTTTGTTTACT	5'RACE product 5'-End (1–675)
**RyR-5'-R2**	ATAAAGCAATGTCCTGTGACCAGAACC	
**RyR-3'-F1**	TAAAGGAGTGCGGGCAGGAGGAGGAA	3'RACE product 3'-End (15 159–15 753)
**RyR-3'-F2**	CCCGACGGAGATGACTACGAAGTTTAT	
**PCRI-F**	TGAATAGCAATTTTGACCGAGCACC	overlapping cDNA fragment PCRI (177–5458)
**PCRI-R**	TGCAGCGATCTTCTTCGGGTATGTG	
**PCRII-F**	CTCTTTCTGCTGCTGTTCTCCCTAC	overlapping cDNA fragment PCRII (5237–10 073)
**PCRII-R**	GTGAGCGTTCCATCAGTTCCTA	
**PCRIII-F**	TTCGTAATCGTCTTAGTGCCTTTG	overlapping cDNA fragment PCRIII (9677–15 626)
**PCRIII-R**	TAACTACCTCCACCACCTCCTGATA	
**ASIa-F**	CAGGTGAAGGCAATGAGGGA	diagnostic PCR for exon a
**ASIa-R**	ATCTAAGAACACGCCGACAAT	
**ASIb-F**	CAGGTGAAGGCAATGAGG	diagnostic PCR for exon b
**ASIb-R**	ACCTTCCTCATACCGAACACC	
**ASII-F1**	CATCTAGTTTACCAAGTGTTTCC	diagnostic PCR for the presence of exon c
**ASII-R1**	ACATCCGTAAGAACGCCAAC	
**ASII-F2**	TACGCAATAAGGTCCGAATA	diagnostic PCR for the absence of exon c
**ASII-R2**	GTTGGGTATTGGTTCTGTAA	
**ASIII-F1**	GCCAATCCAAACCACAAATCAACGA	diagnostic PCR for the presence of exon d
**ASIII-R1**	ACTCCTCCTTTAGATGGCTTGTGTA	
**ASIII-F2**	TCCAAACCACAAATCAGTGAAAGCA	diagnostic PCR for the absence of exon d
**ASIII-R2**	AGTCATAAGGAACAAGTTGAGGAT	
**ASIV-F1**	TACATTCCAAGTGCGGGTGC	diagnostic PCR for the presence of exon e
**ASIV-R1**	AGCGTCCCATAAACGAGAAT	
**ASIV-F2**	TAGCAATACTCCGTGGTGGT	diagnostic PCR for the absence of exon e
**ASIV-R2**	GAACCTACACCAAGACCTTCTGCCT	

### Sequence analysis and phylogenetic tree construction

Nucleotide sequences from individual clones were assembled into a full-length contig using the ContigExpress program, which is part of the Vector NTI Advance 11.5 (Invitrogen) suite of programs. Multiple sequence alignments were performed with DNAMAN v.6.03 (Lynnon Biosoft, USA). The signal peptide was predicted by the SignalP 4.1 server program (http://www.cbs.dtu.dk/services/SignalP). The ExPASy Proteomics Server (http://cn.expasy.org/tools/pi_tool.html) was used to compute the isoelectric point and molecular weight of the deduced protein sequences. Transmembrane region predictions were made using TMHMM 2.0 (http://www.cbs.dtu.dk/services/TMHMM-2.0/). Conserved domains were predicted using the Conserved Domains Database (http://www.ncbi.nlm.nih.gov/cdd). The *RyR* sequences of other species for phylogenetic tree construction were downloaded from GenBank (http://www.ncbi.nlm.nih.gov/Genbank/index.html). The phylogenetic tree was constructed on the basis of amino acid sequences by the software ClustalW [38] and MEGA5.0 [39] using the neighbor-joining method. Bootstrap values were calculated with 1,000 replications.

### Quantitative real-time PCR to investigate mRNA expression levels of *BdRyR*

The relative expression levels of *BdRyR* during various *B. dorsalis*' developmental stages and in different adult body parts were measured using quantitative real-time PCR. Total RNAs were extracted from whole bodies of five insects at each stage, and isolated body parts of adults (pooled from 10 insects), using TRIzol reagent with DNA digestion by DNase (TaKaRa) and the RNeasy Plus Micro Kit (with gDNA Eliminator spin columns, Qiagen, Valencia, CA, USA). First strand cDNA was synthesized from 2 µg of each total RNA preparation in a 10 µl reaction mixture using random hexamer primers and an oligo (dT) primer using the PrimeScript RT reagent Kit (TaKaRa). Real-time PCR was conducted in an Mx3000P thermal cycler (Stratagene, La Jolla, CA, USA) with the stable *B. dorsalis* reference gene *α*-*Tubulin* (GU269902) [40]. The quantitative real-time PCR was carried out in 20 µl reaction volumes containing 1 µl cDNA (200 ng/µl), 10 µl iQ™ SYBR Green Supermix (BIO-RAD, Hercules, CA, USA), 1 µl each of forward and reverse primers (0.2 mM), and 7 µl ddH2O. Primers for BdRyR (forward primer, 5'-GCCTACGGCTACCAGACGAT-3'; reverse primer, 5'-AAGCCAACTGGAAGCAAACG-3', the product located in the conserved domain without deletion site) and for α-Tubulin gene (forward primer, 5'-CGCATTCATGGTTGATAACG-3'; reverse primer, 5'-GGGCACCAAGTTAGTCTGGA-3') were designed using Primer Premier 5.0 software (Premier Biosoft International). Thermocycling conditions were an initial denaturation at 95°C for 2 min; 40 cycles of 95°C for 15 s, 60°C for 30 s, and 72°C for 30 s. After the cycling protocol, the melting curve analysis from 60°C to 95°C was used to verify a single PCR product. Four biological replicates were examined. The amplification efficiencies of the target gene and reference gene were estimated using the equation, E = (10−1/slope)−1, where the slope was derived from the plot of the cycle threshold (Ct) value versus the log of the serially diluted template concentration. The amplification efficiency values for the BdRyR and α-Tubulin were found to be 0.9356 and 1.048, respectively. Quantification of the transcript level of the BdRyR gene was conducted according to the 2−ΔΔCt method [41]. The BdRyR expression data were expressed as means ± standard deviation (SD). A statistical analysis was performed by Duncan's Multiple Range Test for significance (P<0.05) using SPSS 19.0 (SPSS, Inc., Chicago, IL, USA).

### Diagnostic PCR analysis for the detection of alternative exons

Diagnostic PCR was performed to detect the presence of each alternative exon in the individual cDNA clones, according to a previously reported method [23]. The names and sequences of primers for diagnostic PCR analysis are listed in Table 2. Briefly, fragments containing the alternative exons were amplified by four sets of primers flanking the alternative exon region. The PCR products were cloned into pGEM-T Easy vector (Promega), and those positive clones were selected for the diagnostic PCR assay. Mutually exclusive exons (ASI exon a/b) were identified using two exon-specific primers. Optional exons c, d and e, named ASII, ASIII and ASIV, respectively, were identified using a primer spanning the exon and flanking region or a primer spanning the flanking region excluding the exon sequence. These exon-specific primers were paired with counterpart primers located on either side of the alternative spliced segment to generate unique PCR products representing the presence or absence of alternative exons in each clone. The reliability of the diagnostic PCR performed here was further confirmed by sequencing the representative clone that exhibited unique bands.

## Results

### Cloning and analysis of *BdRyR*

The full-length cDNA of *BdRyR* (GenBank accession number: KJ082086) contained a continuous 15,420-bp open reading frame (ORF) sequence, encoding a protein of 5,140 amino acid residues, a 204-bp 5'-UTR with in-frame stop codon, and a 126-bp 3'-UTR with a 32-bp polyA tail. The typical polyadenylation signal, AATAAA, was located 28 bp upstream of the polyadenylation site. The encoded 5,140 amino acid residues predicted a protein with a molecular mass of 582.39 kDa and isoelectric point of 5.38.

**Figure 2:**
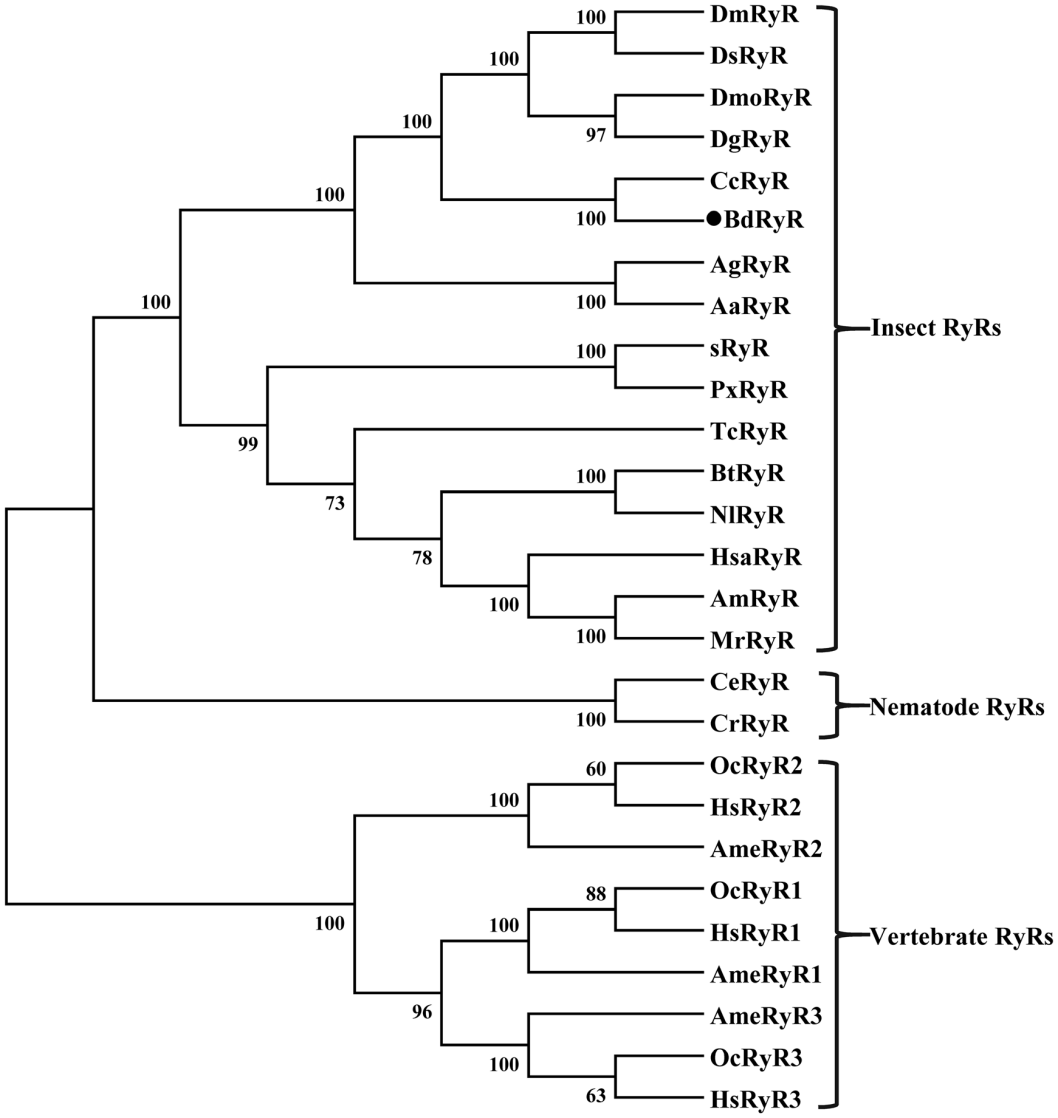
Phylogenetic analysis of the ryanodine receptor (RyR) from insect and non-insect species. The *Bactrocera dorsalis RyR* amino acid sequence was aligned with 26 representative RyR isoforms. The corresponding GenBank accession numbers are as follows: *Drosophila melanogaster* (*DmRyR*), BAA41470; *Drosophila simulans* (*DsRyR*), XP_002080659; *Drosophila mojavensis* (*DmoRyR*), XP_002005714; *Drosophila grimshawi* (*DgRyR*), XP_001995333; *Ceratitis capitata* (*CcRyR*), XP_004527511; *Bactrocera dorsalis* (*BdRyR*), KJ082086; *Anopheles gambiae* (*AgRyR*), XP_318561; *Aedes aegypti* (*AaRyR*), XP_001657320; *Bombyx mori* (*sRyR*), DJ085056; *Plutella xylostella* (*PxRyR*), JN801028; *Tribolium castaneum* (*TcRyR*), EEZ99829; *Bemisia tabaci* (*BtRyR*), AFK84957; *Nilaparvata lugens* (*NlRyR*), KF306296; *Harpegnathos saltator* (*HsaRyR*), EFN78897; *Apis mellifera* (*AmRyR*), XP_392217; *Megachile rotundata* (*MrRyR*), XP_003701507; *Caenorhabditis elegans* (*CeRyR*), BAA08309; *Caenorhabditis remanei* (*CrRyR*), EFP05547; *Ailuropoda melanoleuca* RyR1 (*AmeRyR1*), XP_002925900; *Ailuropoda melanoleuca* RyR2 (*AmeRyR2*), XP_002923703; *Ailuropoda melanoleuca* RyR3 (*AmeRyR3*), XP_002925256; *Homo sapiens* RyR1 (*HsRyR1*), NP_000531; *Homo sapiens* RyR2 (*HsRyR2*), NP_001026; *Homo sapiens* RyR3 (*HsRyR3*), NP_001026; *Oryctolagus cuniculus* RyR1 (*OcRyR1*), NP_001095188; *Oryctolagus cuniculus* RyR2 (*OcRyR2*), NP_001076226; and *Oryctolagus cuniculus* RyR3 (*OcRyR3*), NP_001076231. The neighbor-joining tree was generated in MEGA5.0 with 1,000 bootstrap replicates.

The identities of BdRyR with any one of the three vertebrate RyR isoforms (RyR1 to RyR3) and *Caenorhabditis elegans* were 46 to 47% and 46%, respectively. Identities of BdRyR with RyRs from *Ceratitis capitata*, *D. melanogaster* and *Tribolium castaneum* were 97%, 91%, and 78%, respectively. These scores were significantly higher than those among genetically distinct RyR vertebrate isoforms. To investigate the evolutionary relationships among RyR sequences, a phylogenetic analysis of 20 species' RyR ORFs was conducted by MEGA 5.0 using the neighbor-joining method (Figure 2). According to the phylogenetic tree, the RyRs from 16 insects formed a large cluster, which was well segregated from the RyRs of nematodes and vertebrates. BdRyR was most closely related to CcRyR, with these two genes clustering together.

### Conserved structural domains in BdRyR

**Figure 3:**
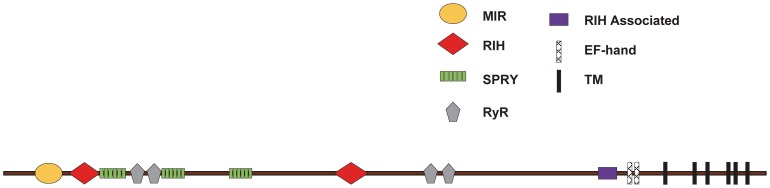
Conserved structural domains in *Bactrocera dorsalis*' ryanodine receptor (*BdRyR*). The relative position of putative transmembrane domains (TM1 to TM6) and conserved structural domains predicted according to the Conserved Domains Database (NCBI) are shown. Those conserved structural domains are listed as follows: MIR (Mannosyltransferase, IP_3_R and RyR) domains (pfam02815), RIH (RyR and IP_3_R Homology) domains (pfam01365), SPRY (splA and RyR) domains (pfam00622), RyR domains (RyR repeated domain) (pfam02026), RIH-associated domains (RyR and IP_3_R Homology associated) (pfam08454), and EF-hands.

Analyses of the amino acid sequence of *BdRyR* indicated that it contained a long N-terminal domain without a signal peptide, which had multiple regulatory domains that modulated the gating of the C-terminal channel pore. The five types of conserved domain (Figure 3) in the N-terminal region of BdRyR included: a mannosyltransferase, IP3R and RyR domain (MIR, pfam02815) at position 211–392; two RyR and IP3R homology domains (RIH, pfam01365) at positions 439–648 and 2227–2456; three spore lysis A (splA) and RyR (SPRY) domains (pfam00622) at positions 665–802, 1092–1215 and 1532–1672; four RyR repeated domains (pfam02026) at positions 853–947, 966–1060, 2833–2926 and 2956–3044; and one RIH-associated domain (pfam08454) at position 4009–4134.

**Figure 4:**
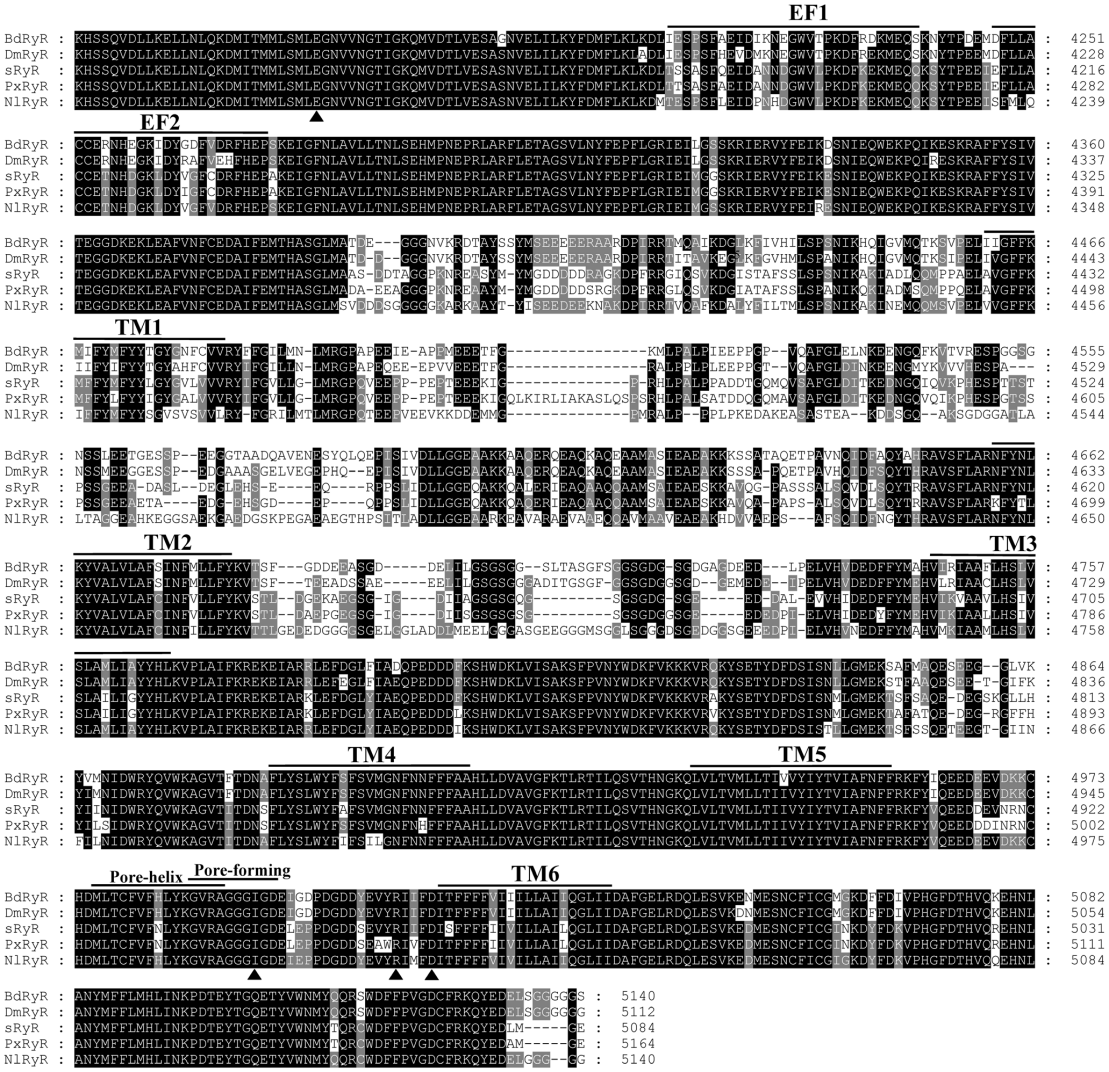
Multiple sequence alignment of the C-terminal region of insect ryanodine receptor (RyR) isoforms. The amino acid position is indicated on the right. Identical residues between BdRyR and any of the aligned sequences are shown as black boxes and similar residues are highlighted in gray boxes. Dashes indicate gaps that have been introduced to maximize homology. The two putative EF-hand motifs (EF1 and EF2), six transmembrane domains (TM1 to TM6), and pore-helix and pore-forming regions are shown using overlining in the figure. Triangles below the alignment indicate the position of important residues (E^4170^, I^4994^, R^5010^ and D^5014^). Abbreviations and GenBank entries for the RyR isoforms are described in Figure 2.

The C-terminal region of RyR protein can form functionally important domains shared by mammalian and insect RyRs [9], [20]. Thus, the C-terminal region of BdRyR was compared with other reported insect RyRs, including DmRyR from *D*. *melanogaster*, sRyR from *B. mori*, PxRyR from *P. xylostella*, and NlRyR from *N. lugens* (Figure 4). A multiple alignment showed that BdRyR contained six hydrophobic transmembrane regions (TM1: 4461–4483, TM2: 4658–4680, TM3: 4746–4768, TM4: 4887–4909, TM5:4935–4957, and TM6: 5015–5034), and these regions exhibited high sequence identities between BdRyR and other insect RyRs. The sequence motif, GVRAGGGIGD, identified between TM5 and TM6, which is known to constitute part of the pore-forming segments of the Ca^2+^ release channels [42], was well conserved in BdRyR (4987–4996) and other insect RyRs. Two EF-hand Ca^2+^ binding motifs originally reported in the lobster RyR [43] were also present in tandem at positions 4210–4238 and 4247–4273. In addition, a glutamate that is proposed to be involved in the Ca^2+^ sensitivity in rabbit RyR1 (E^4032^) [44] and RyR3 (E^3885^) [45] was detected in BdRyR (E^4170^) and other insect RyRs. The residues corresponding to I^4897^, R^4913^, and D^4917^ of rabbit RyR1, which were shown to play an important role in the activity and conductance of the Ca^2+^ release channel [46], were conserved in BdRyR (I^4994^, R^5010^ and D^5014^) and other insect RyRs (Figure 4).

### mRNA expression profiles of *BdRyR*

**Figure 5:**
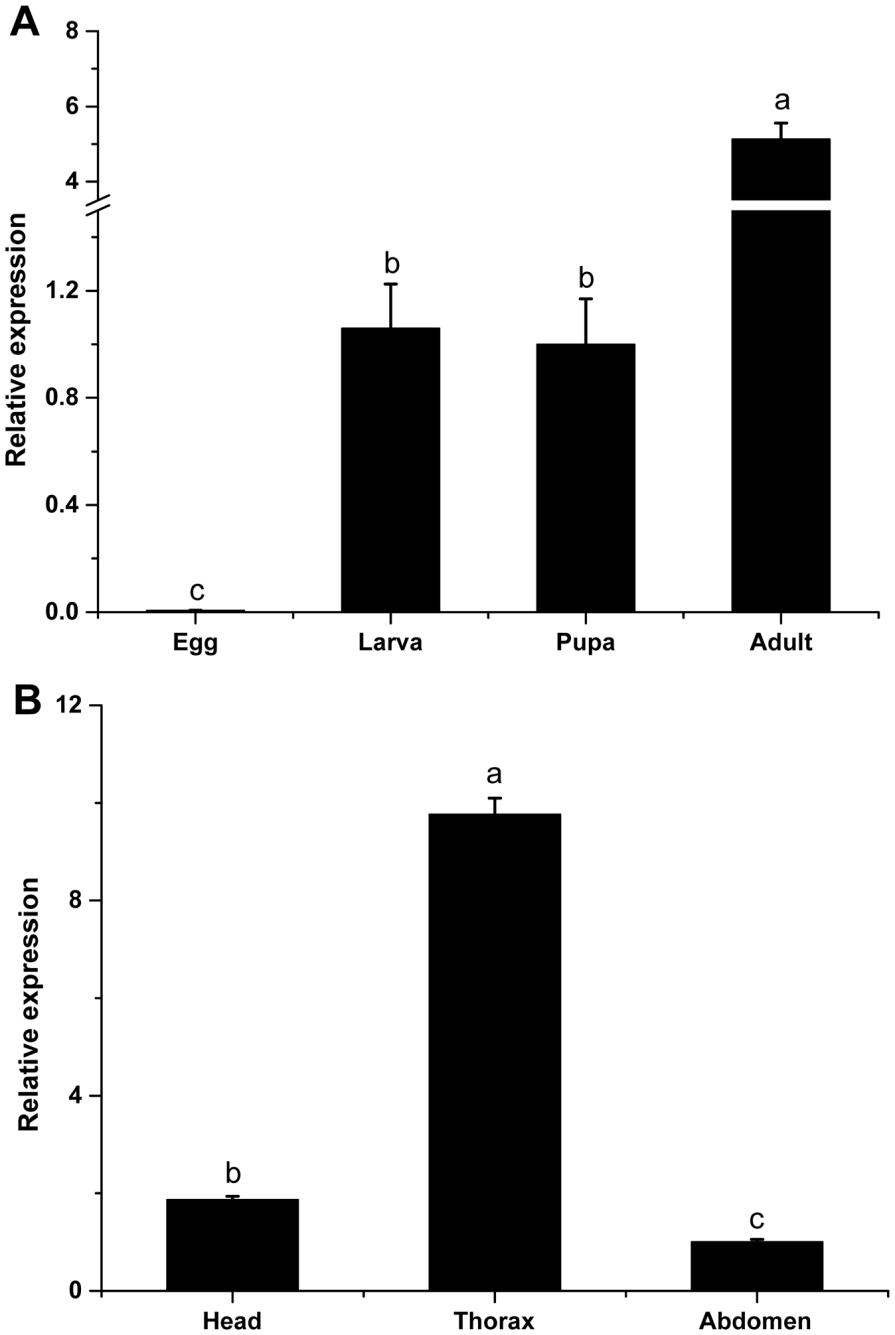
The relative mRNA expression profiles of *Bactrocera dorsalis*' ryanodine receptor (*BdRyR*). A: Expression profiles during different developmental stages including egg, the third instar larva, pupa and adult. B: Expression profiles from different adult body parts including the head, thorax and abdomen. Data are presented as mean ± SE (n = 4). Different letters above each bar indicate statistical difference by ANOVA followed by the Duncan's Multiple Comparison test (*P*<0.05).

The qRT-PCR was performed to investigate the expression levels of *BdRyR* during various *B. dorsalis*' developmental stages (Figure 5 A) and in adult body parts (Figure 5 B). The developmental expression profiles revealed that *BdRyR* was expressed at the lowest and highest levels in egg and adult, respectively (Figure 5 A), and that the *BdRyR* expression levels in the third instar larva, pupa and adult were 166.99-, 157.56- and 808.56-fold higher, respectively, than that in the egg. In the different adult body parts, the highest expression level was observed in the thorax compared with the head and abdomen (Figure 5 B).

### Alternative splicing of *BdRyR*

**Figure 6:**
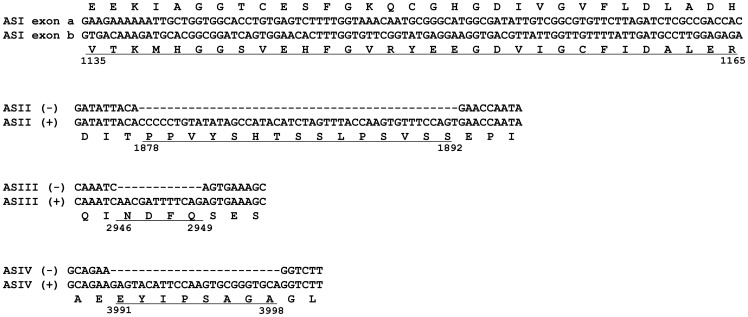
Schematic diagram showing the locations of four alternatively spliced regions of *Bactrocera dorsalis*' ryanodine receptor (*BdRyR*). The cDNA sequences of ASI exon a, ASII (−), ASIII (−) and ASIV (−) are aligned with those of ASI exon b, ASII (+), ASIII (+) and ASIV (+), respectively. The corresponding amino acid residues are shown below (above for ASI exon a) the cDNA sequences in a single-letter code. Underlined sequence indicate the two mutually exclusive exons a and b, and residues absent in ASII (−), ASIII (−) and ASIV (−). Amino acid residues at splice sites are numbered.

The sequence analysis of multiple *BdRyR* cDNA clones identified four regions that showed heterogeneity. The first one was located between amino acid residues 1135 and 1165, generating two mutually exclusive exons, ASI exons a and b, which were highly divergent, differing at 20 of 31 amino acid residues. For the other three regions, the inclusion and exclusion of 45-bp (ASII), 12-bp (ASIII) and 24-bp (ASIV) segments generated two variants ASII (+) and ASII (−), ASIII (+) and ASIII (−), ASIV (+) and ASIV (−), respectively. The analysis of the locations of the four alternative spliced regions of *BdRyR* revealed that ASI was located in the central part of the predicted second SPRY domain, ASII was located between the third SPRY domain and the second RIH domain, ASIII was located between the third RyR and the fourth RyR domain, and ASIV was located near the RIH-associated domain (Figure 6).

**Figure 7:**
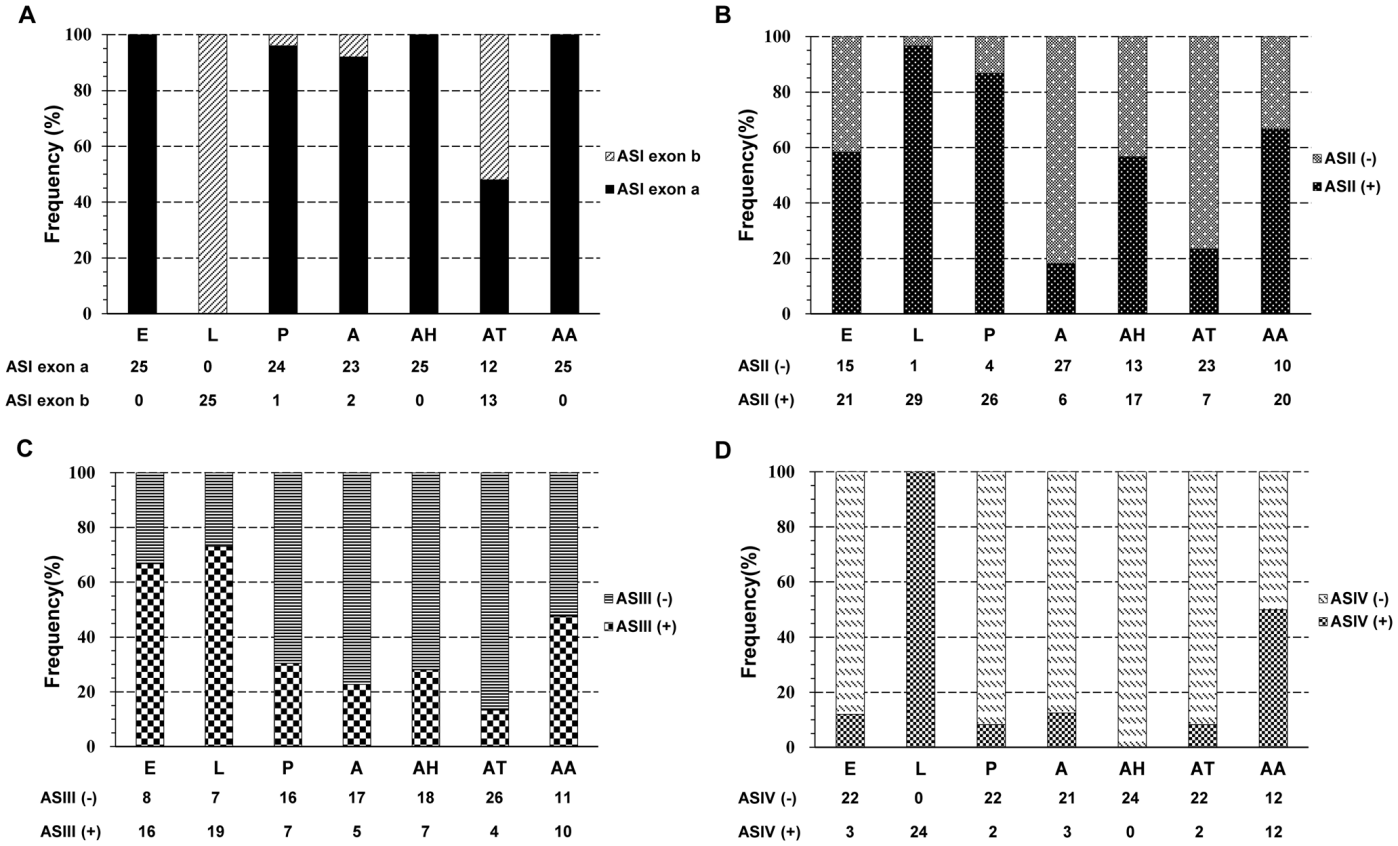
The relative frequencies of *Bactrocera dorsalis*' ryanodine receptors (*BdRyR*) mutually exclusive exon (A) and optional exon (B, C and D) in various developmental stages and different adult body parts. E (egg), L (larva), P (pupa), A (adult), AH (adult head), AT (adult thorax) and AA (adult abdomen). The number of clones for each alternative splice variant detected by diagnostic PCR is listed.

The presence of each putative alternative splice variant in seven discrete mRNA pools (egg, larva, pupa, adult, adult head, adult thorax, and adult abdomen) was determined by diagnostic PCR (Figure 7). Data were collected from sets of 21 to 36 clones for each developmental stage and body part. Mutually exclusive ASI exons a and b exhibited significant developmental and anatomical regulation. ASI exon a was prominently present in cDNA clones from most developmental stages and body parts, with the exception of larva, where no ASI exon a was detected, and the adult thorax, where only 12 in 25 clones possessed ASI. ASI exon b was not detected in egg, adult head and adult abdomen, while it was present at low to high frequencies, 4%, 8%, 52% and 100%, in the pupa, adult, adult thorax and larval cDNA pools, respectively. The developmental and anatomical regulations of ASII, ASIII and ASIV were also observed. ASII (+) was detected at low frequencies, 18% and 23%, in adult and adult thorax, while at moderate to high frequencies, 57%, 58%, 67%, 87% and 97%, in adult head, egg, adult abdomen, pupa and larva, respectively. ASIII (−) and ASIV (−) were dominant in most developmental stages and body parts compared with ASIII (+) and ASIV (+), respectively, with the exception of ASIII (−) in egg (33%) and larva (27%), and ASIV (−) in larva (0%).

## Discussion

Studies on the RyRs of insects have become increasingly intensive with the emergence of diamide insecticides, which were recently developed and show a high selective toxicity against insects compared with mammals. With the first description of insect RyR from *D. melanogaster* in 1994 [47], insect RyRs have been isolated and characterized in various insect species, such as *B*. *mori* (*sRyR*), *P*. *xylostella* (*PxRyR*), *C*. *medinalis* (*CmRyR*), *N. lugens* (*NlRyR*), *O. furnacalis* (*OfRyR*), *H. armigera* (*HaRyR*), and *P. rapae* (*PrRyR*) [21]–[27]. In the present study, we identified and sequenced the full-length RyR cDNA (named *BdRyR*) from *B. dorsalis*. The deduced 5,140 amino acid residues of *BdRyR* have a high degree of identity (79 to 97%) with other known insect RyRs, while sharing a low degree of identity (46 to 47%) with vertebrate RyR isoforms. The amino acid motif GXRXGGGXGD is critical for ryanodine binding and ion conduction and probably constitutes the pore-forming segment of RyR [42]. This type of motif (GVRAGGGIGD) was found in BdRyR and is conserved in other insect RyRs. Two Ca^2+^ binding EF-hand motifs of BdRyR are present, and these motifs may be involved in the Ca^2+^-dependent inactivation of the RyR in lobsters and mammals [43]. BdRyR also contains several conserved domains, including the RyR, the MIR and RIH domains, which are unique to RyR channels and conserved in all members of the intracellular Ca^2+^-release channel superfamily [48], [49]. Moreover, the phylogenetic analysis revealed that BdRyR is distantly related to the vertebrate and nematode RyRs, while closely related to other insect RyRs. This confirms that the cloned cDNA encode RyR isoforms. Taken together, these results indicate that BdRyR is a structural and functional analog of other known RyRs.

Although diamide insecticides, such as flubendiamide and chlorantraniliprole, show a high selectivity for insect RyRs, their exact binding sites are not yet clear. The binding studies on microsomal membranes from *Heliothis virescens* and *Periplaneta americana* muscles suggested that flubendiamide and chlorantraniliprole interacted with a site distinct from the ryanodine binding site on the insect RyR complex [50], [51]. Subsequently, radioligand binding studies conducted on house fly muscle membranes suggested that flubendiamide and chlorantraniliprole bound at different allosterically coupled RyR sites [52]. The interaction of flubendiamide with the recombinant silkworm RyR (sRyR) suggested that flubendiamide induced Ca^2+^ release through the sRyR by acting on the transmembrane domain (residues 4111–5084), and that the flubendiamide response of sRyR requires the N-terminal cytoplasmic domain (183–290) [21]. Recently, a 46 amino acid segment in the C-terminal transmembrane region of DmRyR (residues 4610–4655) was proven to be critical for diamide insecticide sensitivity [53]. Additionally, an amino acid replacement (G4946E) in the C-terminal membrane-spanning domain of the PxRyR was associated with diamide cross-resistance in *P*. *xylostella* collected from the Philippines and Thailand [30]. Subsequently, it was experimentally proven that the G4946E mutation in PxRyR conferred resistance to chlorantraniliprole in *P*. *xylostella* [32].

In the present study, four residues believed to be related to Ca^2+^ stimulation were conserved in BdRyR. Three of these residues, I^4994^, R^5010^ and D^5014^, were between transmembrane 5 and 6, suggesting that BdRyR may constitute a functional Ca^2+^ release channel similar in basic behavior to those of documented insect RyRs. The amino acid alignment (Figure S1) showed that the fragment of BdRyR (residues 182–289) had a high sequence identity with DmRyR (96%) and sRyR (94%), and a moderate sequence identity (48% to 51%) with three rabbit RyR isoforms. A fragment of the C-terminal transmembrane region of BdRyR (residues 4620–4666) (Figure S2) showed 87% and 62% identity with DmRyR and sRyR, respectively, and 40% to 50% identity with three rabbit RyR isoforms. To some extent, these results could explain the highly selective toxicity of diamide insecticides to insects and mammals. Since G4946E replacement (corresponding to G4917 in BdRyR) in PxRyR was associated with diamide insensitivity in *P. xylostella*, we proposed that the C-terminal transmembrane domain was critical for the selective toxicity of diamide insecticides. Furthermore, the fragment (residues 4620–4666 in BdRyR) in the C-terminal transmembrane region might contribute to the selective toxicity of diamide insecticides against insect and mammalian RyRs.

The differential expression levels of genes in different developmental stages or body parts suggested that different functions might be involved. In the present study, the mRNA expression levels of *BdRyR* in larva, pupa and adult were significantly higher than in egg. A similar result was also reported in *H*. *armigera* [26]. In adults, the expression level of *BdRyR* in the thorax was much higher than that in the head or abdomen, which was consistent with the report in *P. rapae* [27]. These results indicate that RyR modulates the balance of the calcium concentration in the locomotive system. The adult and larva are more active than the egg, and the adult thorax is considered to be the major muscle tissue responding to the insect's locomotion.

Alternative splicing is a major contributor to transcriptomic and proteomic complexity, disease, and development. Alternative splicing may affect the protein sequence in two ways: (i) by deleting or inserting a sequence and creating long and short isoforms, or (ii) by substituting one segment of the amino acid sequence for another [54]. It was reported that 12 distinct splice variants had been identified in RyR isoforms cloned from mammals [55]. The expressions of RyR splice variants in mammals are regulated both developmentally and in a tissue-specific manner among RyR isoforms [55]–[58], and some splice variants can suppress intracellular Ca^2+^ fluxes or exhibit distinct Ca^2+^-releasing profiles [10], [59], [60]. In contrast to mammals, only one RyR gene was found in insects, which showed only a 44 to 46% amino acid identity with the three mammalian RyRs [20]. Hence, the diversity of insect RyRs could be increased through alternative splicing. Two alternative splice sites of RyR were first found in *D*. *melanogaster* [47], and subsequently, similar splicing patterns were also reported in a series of agricultural pests [23]–[26]. In the present study, four alternative splice sites were found in the *BdRyR* gene. Three alternative splice sites, ASI, ASII and ASIV, were conserved from *D*. *melanogaster* [20], [40], but one, ASIII, was reported for the first time. Like the alternative splice variants reported in mammals, the four variants in *BdRyR* exhibited significant developmental or anatomical regulation. Remarkably, ASI (1135–1165) was located in the central part of the predicted second SPRY domain. The SPRY domain was identified as a region of homology in a non-receptor tyrosine kinase of *Dictyostelium discoideum*, splA, and in the mammalian RyR calcium-release channels [61]. To date, the majority of functional analyses have implicated them in protein-protein interactions. The second of three SPRY domains in RyR1 interacts intramolecularly with the alternatively spliced residues and neighboring basic residues of RyR1 to regulate excitation coupling in skeletal muscles [62]. Since there is a divergence of residues between ASI exons a and b, it might possess various functions. No ASI exon a was detected in the larval stage of *B*. *dorsalis*, while exons equivalent to exon a in *BdRyR* were predominantly detected in the larval/nymph stage of *C*. *medinalis*, *N*. *lugens*, and *O*. *furnacalis* [23]–[25]. Thus, the splice variants of RyR might vary in different insect species. However, the highest frequencies of ASII (+), ASIII (+) and ASIV (+) were detected in the larval stage. The above results indicated that developmentally regulated alternative splicing might play an important role in the differential properties of the Ca^2+^ release channel. Further studies are needed to elucidate the properties and functions of each *BdRyR* splice variant using various strategies, such as *in vitro* expression, ligand binding analysis, and electrophysiological experiments.

In summary, we first isolated and characterized the RyR gene from *B*. *dorsalis*, and investigated the frequencies of alternative splice variants in various *B*. *dorsalis*' developmental stages and different adult body parts, which is an important step and lays the foundation for understanding its structural and functional properties, and the molecular mechanisms for target site resistance in *B*. *dorsalis*.

## Supporting Information

Figure S1 **Multiple alignments of the N-terminal region of** ***Bactrocera dorsalis*** **' ryanodine receptor (** ***BdRyR*** **) with other representative insect and mammal RyRs.** Identical amino acids and similar amino acids are shown in black boxes and gray boxes, respectively. Dashes indicate gaps that have been introduced to maximize homology. Abbreviation and GenBank entries for the RyR isoforms are described in Figure 2.
(TIF)

Figure S2 **Multiple alignments of the C-terminal transmembrane region of** ***Bactrocera dorsalis*** **' ryanodine receptor (** ***BdRyR*** **) with other representative insect and mammal RyRs.** Identical amino acids and similar amino acids are shown in black boxes and gray boxes, respectively. Dashes indicate gaps that have been introduced to maximize homology. Abbreviation and GenBank entries for the RyR isoforms are described in Figure 2.
(TIF)
