# Supplementary figures and images for: Molecular Characteristics, mRNA Expression, and Alternative Splicing of a Ryanodine Receptor Gene in the Oriental Fruit Fly, Bactrocera dorsalis (Hendel)
Source: PLoS One. 2014 Apr 16;9(4):e95199. doi: 10.1371/journal.pone.0095199 (PMC3989282; doi:10.1371/journal.pone.0095199)

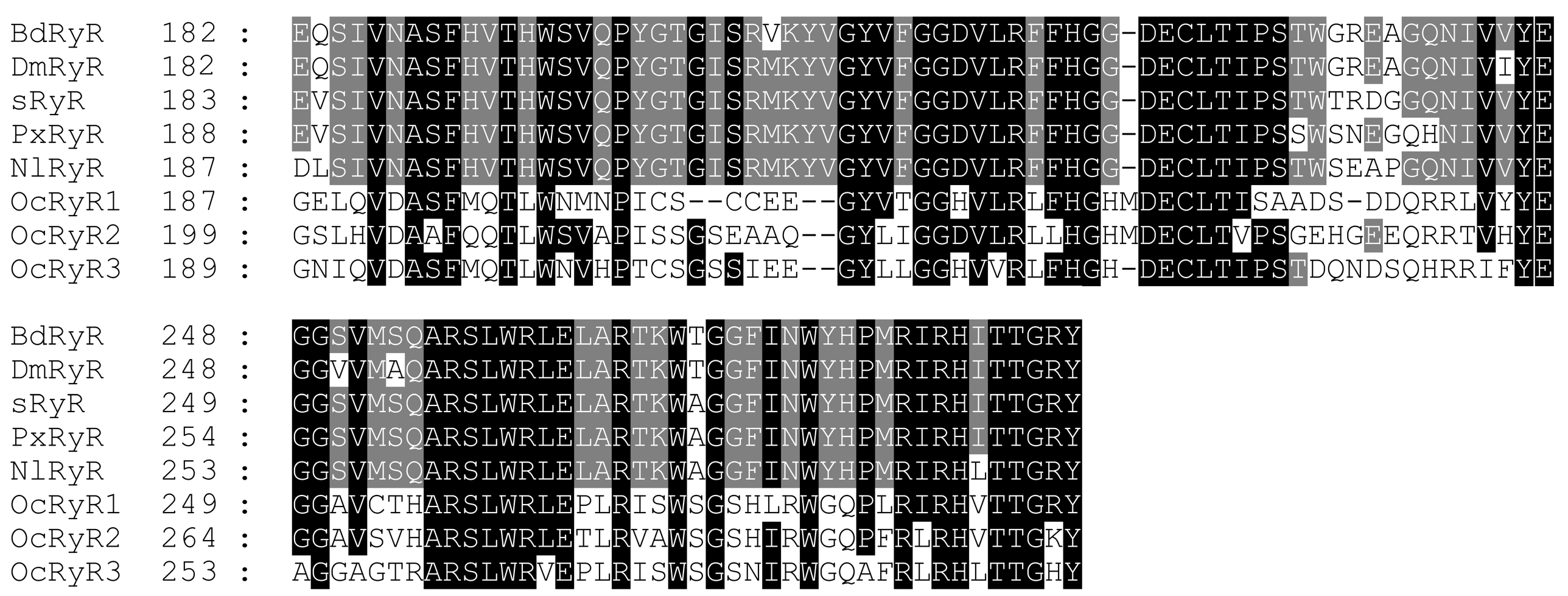

Supplement: Figure S1 — Multiple alignments of the N-terminal region of Bactrocera dorsalis ' ryanodine receptor ( BdRyR ) with other representative insect and mammal RyRs. Identical amino acids and similar amino acids are shown in black boxes and gray boxes, respectively. Dashes indicate gaps that have been introduced to maximize homology. Abbreviation and GenBank entries for the RyR isoforms are described in Figure 2. (TIF) [file pone.0095199.s001.tif]

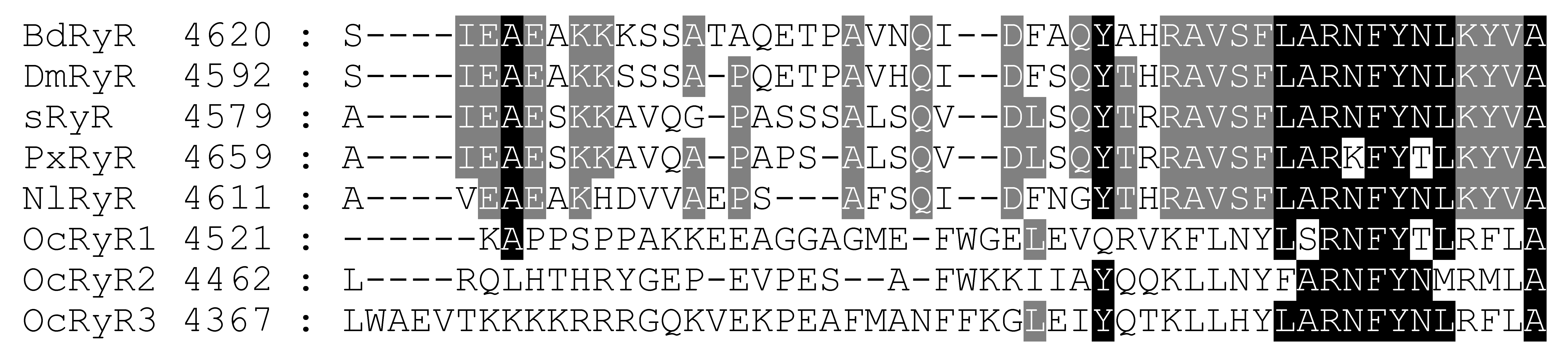

Supplement: Figure S2 — Multiple alignments of the C-terminal transmembrane region of Bactrocera dorsalis ' ryanodine receptor ( BdRyR ) with other representative insect and mammal RyRs. Identical amino acids and similar amino acids are shown in black boxes and gray boxes, respectively. Dashes indicate gaps that have been introduced to maximize homology. Abbreviation and GenBank entries for the RyR isoforms are described in Figure 2. (TIF) [file pone.0095199.s002.tif]
